# Holocene ice marginal fluctuations of the Qassimiut lobe in South Greenland

**DOI:** 10.1038/srep22362

**Published:** 2016-03-04

**Authors:** Nicolaj K. Larsen, Jesper Find, Anders Kristensen, Anders A. Bjørk, Kristian K. Kjeldsen, Bent V. Odgaard, Jesper Olsen, Kurt H. Kjær

**Affiliations:** 1 Department of Geoscience, Aarhus University, Høegh Guldbergs Gade 2, 8000 Aarhus C, Denmark; 2 Centre for GeoGenetics, Natural History Museum of Denmark, University of Copenhagen, Øster Voldgade 5-7, 1350 Copenhagen K, Denmark; 3 Department of Physics and Astronomy, Aarhus University, 8000 Aarhus C, Denmark

## Abstract

Knowledge about the Holocene evolution of the Greenland ice sheet (GrIS) is important to put the recent observations of ice loss into a longer-term perspective. In this study, we use six new threshold lake records supplemented with two existing lake records to reconstruct the Holocene ice marginal fluctuations of the Qassimiut lobe (QL) – one of the most dynamic parts of the GrIS in South Greenland. Times when the ice margin was close to present extent are characterized by clastic input from the glacier meltwater, whereas periods when the ice margin was behind its present day extent comprise organic-rich sediments. We find that the overall pattern suggests that the central part of the ice lobe in low-lying areas experienced the most prolonged ice retreat from ~9–0.4 cal. ka BP, whereas the more distal parts of the ice lobe at higher elevation re-advanced and remained close to the present extent during the Neoglacial between ~4.4 and 1.8 cal. ka BP. These results demonstrate that the QL was primarily driven by Holocene climate changes, but also emphasises the role of local topography on the ice marginal fluctuations.

In South Greenland the 100 km wide Qassimiut lobe (QL) is presently one of the most climate sensitive parts of the Greenland ice sheet (GrIS)^1^ and it also experienced notably large changes during the Holocene^2^. The QL consists of several relatively fast-flowing marine terminating outlet glaciers intersected by more passive landbased ice^2^ (Fig. 1). The central proximal part of the ice lobe is situated below 500 m a.s.l. and the more distal parts (flanks) of the ice lobe lie between c. 900 m–1500 m a.s.l. Ice loss of the QL is dominated by melting but calving ice production is appreciable at a number of the marine terminating glaciers^3^. During the Last Glacial Maximum (LGM)^4^ the GrIS extended to the shelf break in South Greenland and it began to disintegrate around ~19–18 cal. ka BP^5^,^6^,^7^. By ~14.1 cal. ka BP the southern tip of Greenland was ice-free and the outer part of Ikersuaq Fjord at Julianehåb Bay was deglaciated slightly later at ~12 ka^8^,^9^,^10^. The ice margin reached the present extent by ~11.1–10.5 ka^11^,^12^. Radiocarbon ages of reworked organic molluscs in Little Ice Age (LIA) moraines suggest that the ice margin of the QL experienced large fluctuations during the Holocene^2^ and sediment cores from the ice marginal Qipisarqo lake reveal that the central part of the QL was retracted behind present day extent from ~9.1–0.4 cal. ka BP^13^. In contrast, the ice margin to the east retreated behind its present extent ~6.9 cal. ka BP and readvanced at ~3–2.8 cal. ka BP and ~0.4 cal. ka BP during the subsequent LIA^11^. Previous research has concluded that the response of the QL was mainly governed by climatic changes and that the differences of ice marginal response were related to the local topographic setting between the two threshold lake sites^11^. However, it is not clear to what extent local topography influences the ice marginal behaviour and this warrants further investigation. Here, we present new constraints on how the QL responded to Holocene climate forcing mechanisms.

## Results–Core Description and Interpretation

In this study, we use proglacial threshold lakes to constrain the ice marginal fluctuations of the QL. Threshold lakes are located adjacent to the ice margin and by analysing the sediment cores it is possible to determine when the glaciers are present within the lake catchment (i.e. similar ice extent as today) and periods where the ice margin was retracted and significantly smaller than today^14^. These changes are clearly seen in the sediment cores as changes from clastic to organic-rich sediment (see methods). We cored and analysed six new threshold lakes (Figs 2, 3, 4, 5); two are from the central part of the lobe (Sermilik, Kangerdluatsiaup tasia), three are from the margin of the ice lobe (Akuliarusseq, Storesø, Rundesø) and one is located 50 km northwest of the QL (Kingigtoq). These new results will, together with the existing data from South Greenland, give a more coherent picture of the Holocene ice marginal fluctuations of the QL.

### Kingigtoq lake

**Figure 1:**
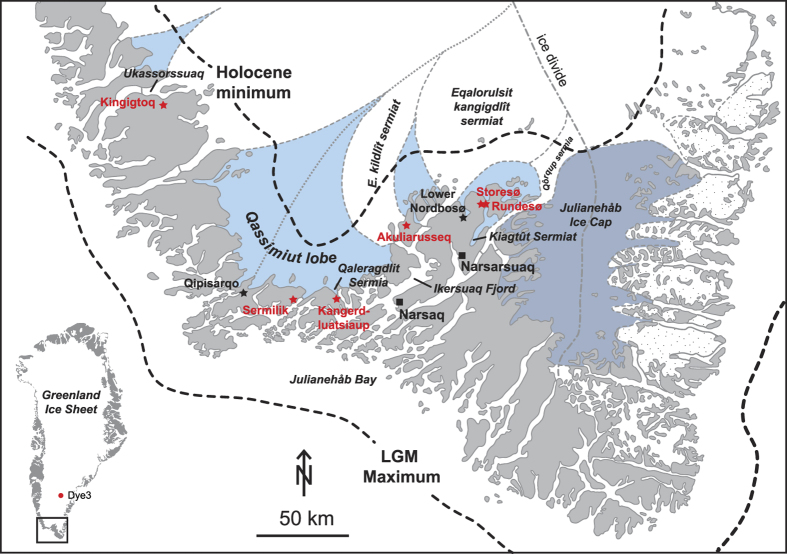
Overview map of the study area with the subdivision of the Greenland ice sheet, and local ice caps^42^. Active areas of the Greenland ice sheet are marked in light blue and passive areas are marked as white areas. New and existing threshold lakes are marked with red and black stars, respectively (Adobe Illustrator CS6 was used to create this map; https://www.adobe.com).

**Figure 2:**
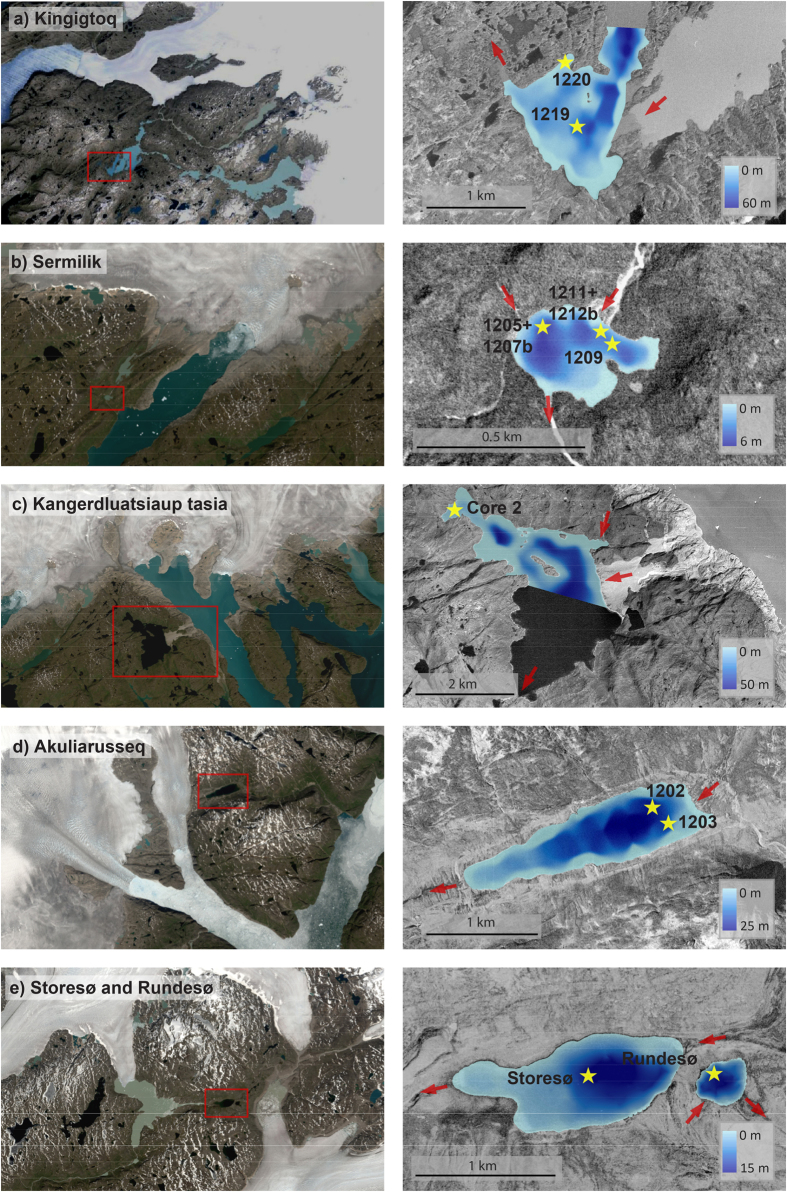
Detailed location and bathymetric maps of the threshold lakes analysed in this study showing the relationship of the lake and the ice sheet. Red arrows mark inflow and outflow of the lakes Overview maps (left hand) are derived from Landsat 8 satellite imagery (http://earthexplorer.usgs.gov/). Orthophotos (right hand) are derived using aero-triangulated vertical stereo photogrammetric imagery recorded in 1985 (Ref. 1). (Maps were created in ESRI’s ArcGIS ver. 10.3; http://www.esri.com). All images are orientated towards true north.

**Figure 3:**
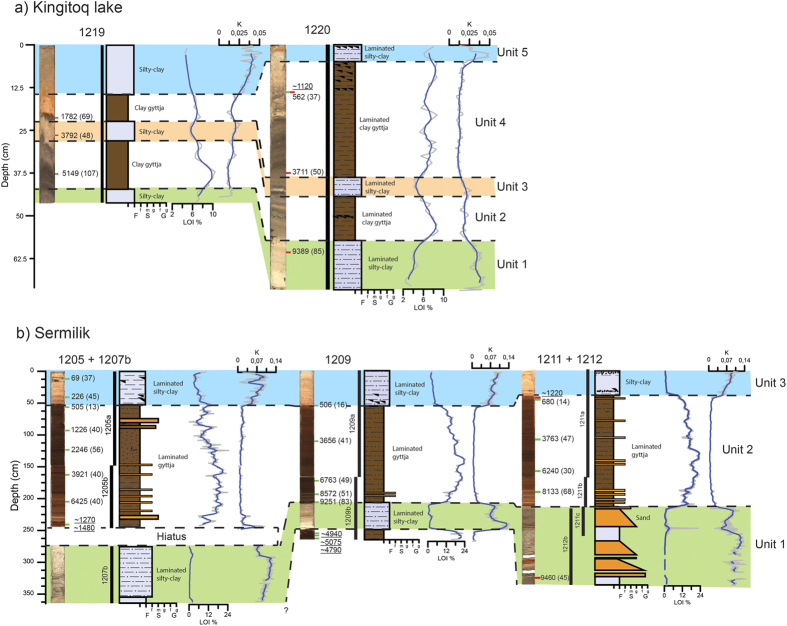
Sediment proxies (core images, stratigraphy, ^14^C ages, LOI and XRF K-counts) from: (**a**) Kingigtoq subdivided into five units. Note that the cores images appear darker due to the sediments oxidizing and (**b**) Sermilik cores subdivided into three units. The green marks on the cores represent radiocarbon ages based on terrestrial macrofossils, whereas red marks represent bulk radiocarbon ages. Note that the underlined radiocarbon ages from the lowermost part of cores 1205 and 1209 show reversals and are therefore not used in the age models (see main text).

Kingigtoq lake is located 550 m a.s.l. near the Ukassorssuaq glacier c. 30 km from the present ice margin. It presently receives meltwater from outlet glaciers that terminate on land and it has to retreat an unknown distance (km’s) before the meltwater inflow ceases (Fig. 1). The lake is 6 km long and 3.2 km wide and consists of several sub-basins up to c. 60 m deep (Fig. 2a). Two cores were retrieved from the southern part of one of the sub-basins – one from the central part at 33 m water depth (Core 1219) and one close to the shore at 6 m water depth (Core 1220). Cores 1219 and 1220 have been correlated and subdivided into five sub-units – three clay units 1, 3, 5 interlayered with clay-gyttja units 2, 4 (Fig. 3a). The age models from the two cores (1219, 1220) are based on six bulk ages and one macrofossil age (Fig. 6). At 14 cm a paired terrestrial macrofossil and bulk age suggest an offset of 650 ± 33 years and this offset has been used in the age models for both cores. In addition, a number of links have been made between the two cores at the distinct clay units at 41 and 4 cm in core 1219 and 1220, respectively.

The clay units are interpreted as representing periods with glacial meltwater input to the lake whereas the clay-gyttja units reflect periods where the ice margin is retracted behind the present extent. The two age models are considered reliable although the timing of the middle clay unit, unit 3, is somewhat uncertain. Based on the age model, the lake was deglaciated before 9.5 cal. ka BP. Meltwater inflow occurred until ~9 cal. ka BP and again from ~4.5 to 3 cal. ka BP and was interrupted by a period of clay-gyttja deposition although the timing is uncertain because of the poorly constrained age model at this interval (Fig. 7). The most recent inflow of meltwater began ~0.3 cal. ka BP during the LIA. In between the periods with meltwater inflow the ice margin was retracted and clay-gyttja was deposited.

### Sermilik lake

Sermilik lake is located 100 m a.s.l. in a small depression, 4 km from the present ice margin at the central part of the QL (Fig. 1). The lake currently receives meltwater and the ice margin has to retreat at least several kilometres before the meltwater inflow to Sermilik lake ceases (Fig. 2b). The lake is 400 m wide and 200 m long and it consists of several smaller basins up to 6 m deep. Cores from three sites were retrieved from the northern part of the lake: 1205 and 1207b (overlapping cores) at 4.4 m water depth and 1209, 1211, 1212 (1211 and 1212 are overlapping cores) at 4.2 m water depth. The cores have been correlated and subdivided into three units: a lower unit composed of silty-clay (core 1212 has more sandy sediments); a middle unit composed of gyttja with several distinct mm-to-cm thick layers of sand; and an upper unit composed of silty-clay (Fig. 3b). In addition, there is a thin layer of gyttja below unit 1 in core 1209. The radiocarbon ages of the gyttja are younger than the overlaying sediments and therefore the gyttja layer is believed to be a coring artifact caused when the corer accidently was lowered back into the sediments when it was pulled-up. We hypothesise the same issues occurred when retrieving core 1205, where three radiocarbon ages yield consistently younger ages than the succession above. Therefore we do not consider the results from these units of the cores in the following interpretation and discussion. The age models are based on twenty-one macrofossil and three bulk radiocarbon ages. At two intervals, paired bulk and macrofossil ages reveal an average offset of 516 ± 27 years and this has been used to correct for the lowermost bulk age at 330 cm.

The clayey-silt units 1 and 3 are interpreted as representing periods with glacial meltwater input to the lake, whereas gyttja unit 2 reflects a period where the ice margin was retracted behind the present extent. The thin sand layers in gyttja unit 2 (1205 and 1211) are interpreted as debris or grain flows formed close to the inlets of the lake where deltas have formed and not as evidence of presence of glacier ice in the catchment. According to the well-constrained age models (Fig. 6), the lake was deglaciated before ~9.6 cal. ka BP and the meltwater inflow continued until ~9.4 cal. ka BP. Meltwater inflow began again at ~0.5 cal. ka BP (Fig. 7).

### Kangerdluatsiaup tasia

**Figure 4:**
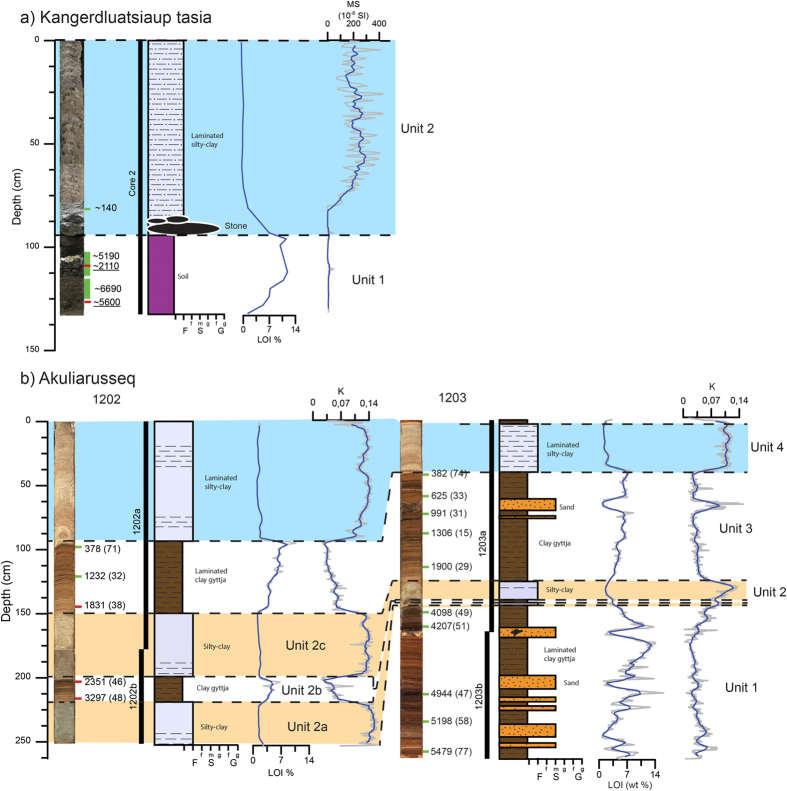
Sediment proxies (core images, stratigraphy, ^14^C ages, LOI and XRF K-counts) from: (**a**) Kangerdluatsiaup tasia subdivided into two units and (**b**) Akuliarusseq subdivided into four units. The green marks on the cores represent radiocarbon ages based on terrestrial macrofossils whereas red marks represent bulk radiocarbon ages. Note that underlined radiocarbon ages are not used in the age model.

Kangerdluatsiaup tasia (lake) is located in the foreland of the central part of the QL adjacent to the Qaleragdlit Sermia (glacier) that terminates in a fjord connected to Ikersuaq Fjord (Fig. 1). During the LIA the Qaleragdlit Sermia advanced more than 8 km from its present extent and dammed a valley forming the Kangerdluatsiaup tasia 100 m a.s.l.^15^. After the glacier retreated, the lake remained dammed by the sediments that were deposited during the LIA. Sediment outcrops reveal 15–20 m large delta foresets consisting of sand and gravel below topset sediments (braided river) in the proximal part of the lake close to the fjord. The lake is 4.4 km long and 2.7 km wide and it consists of several sub-basins up to 50 m deep (Fig. 2c). It was not possible to record lake bathymetry in the southern part of the basin because of lake-ice. One core was retrieved and analysed from a sub-basin in the northwestern part of the lake at 7.7 m water depth. The core consists of a lower unit of sandy sediments with pebbles and small stones and an upper unit of laminated clay (Fig. 4a). The lamina consists of mm-to-cm thick alternating silty-clay and silty-sand layers, which are clearly expressed in the magnetic susceptibility data. Little organic material is present in the core, particularly in the upper clay unit. However, four macrofossil and bulk samples from the lower unit and one macrofossil sample from the upper unit have been dated (Fig. 4a).

The lower unit is interpreted as a soil covering the valley floor whereas the laminated clay was deposited when the valley was dammed by the Qaleragdlit Sermia and meltwater flowed into the valley and deposited foresets and topsets in the proximal part of the lake and bottomsets in the distal part. The bulk and macrofossil ages of the soil are not in chronological order and range from ~2.1–6.7 cal. ka BP (Fig. 4a). However, we consider the two macrofossil ages most reliable, which suggest an age of soil formation between ~5.2 and 6.7 cal. ka BP. We interpret the clay deposition on the valley floor soil to represent a disconformity and therefore do not use those ages in the age model. Instead, we use the youngest radiocarbon age of macrofossil within the clay as a minimum limiting age and conclude that the meltwater inflow began >0.14 cal. ka BP during the LIA (Fig. 7).

### Akuliarusseq lake

Akuliarusseq lake is located adjacent to the outlet glacier that terminates in the Ikersuaq fjord in South Greenland (Fig. 1). It receives meltwater when the ice margin is located close to the LIA limit, but at present there is no inflow of meltwater to the lake. Echo soundings reveal that the elongate lake consists of two basins separated by the remnants of an end-moraine that acts a deep sill (Fig. 2d). Two cores, 1202 and 1203, were retrieved at 20.6 and 17.3 m water depth, respectively. The two cores have been correlated and subdivided into four units (Fig. 4b). The changes between the units of gyttja and clay are very distinct in the cores and the lower boundary between the gyttja and clay is sharp, whereas the boundary between clay and gyttja is transitional. The lower unit 1 only occurs in core 1203 and consists of gyttja with distinct 2–10 cm thick sand layers. Unit 2 has been subdivided into three units and consists of clay interlayered with 2–15 cm gyttja. Unit 3 comprises laminated gyttja followed by unit 4 consisting of massive to laminated clay. The age model of Akuliarusseq lake is based on fifteen radiocarbon samples of which three are bulk samples (Fig. 6). The bulk samples appear in good agreement with the terrestrial radiocarbon ages and hence no offset correction is applied. Clay bands based on lithology and Potassium (K counts) in the two cores have been linked during age modelling at depths 211, 189, 141 and 89 in core 1203 with depth 145, 139, 127 and 41 cm in core 1202. The lower part of core 1202 has not been dated because of absence of organic material in the clay. The two samples from the first gyttja layer (unit 2B) are bulk ages, and therefore we have more confidence in the age model from core 1203. Accordingly, we base our conclusion mainly on the age model from core 1203 in the lower part and use both age models in the upper part of the cores where they agree very well.

The clayey-silt units are interpreted as representing periods of glacial meltwater input to the lake, whereas the gyttja units reflect periods where the ice margin was retracted behind the present extent. The distinct sand layers in the gyttja in core 1203, which is located close to a small inlet in the most distal part of the lake, are interpreted as sediment debris flow deposits and not as evidence of presence of glacier ice in the catchment. Based on visual inspection and the sediment proxies, there is good correlation between the upper and the lower clayey-silt unit in the two cores. Deglaciation of the lake basin is not recorded in the cores because the core bottoms out in gyttja with a basal age of ~5.5 cal. ka BP (Fig. 7). Meltwater inflow was recorded during three events: ~3.4–3.0 cal. ka BP, 2.5–1.9 cal. ka BP and again from ~0.4 cal. ka BP until the most recent ice retreat, which has left a thin layer of gyttja at the top of core 1203.

### Storesø og Rundesø

**Figure 5:**
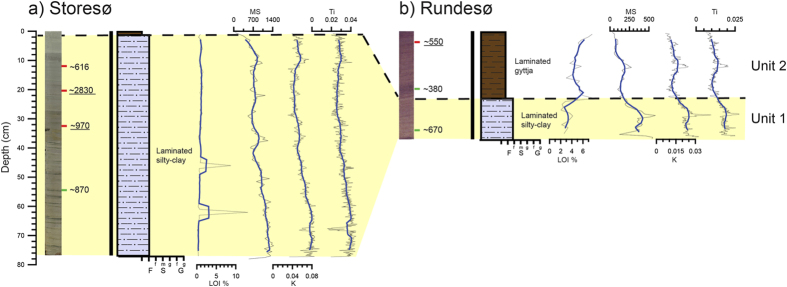
Sediment proxies (core images, stratigraphy, ^14^C ages, LOI, Magnetic susceptibility, and XRF K and Ti-counts) from: (**a**) Storesø and (**b**) Rundesø which both are subdivided into two units. The green marks on the cores represent radiocarbon ages based on terrestrial macrofossils whereas red marks represent bulk radiocarbon ages. Note that underlined radiocarbon ages of bulk sediments are considered to be to old and are not used in the analysis.

**Figure 6:**
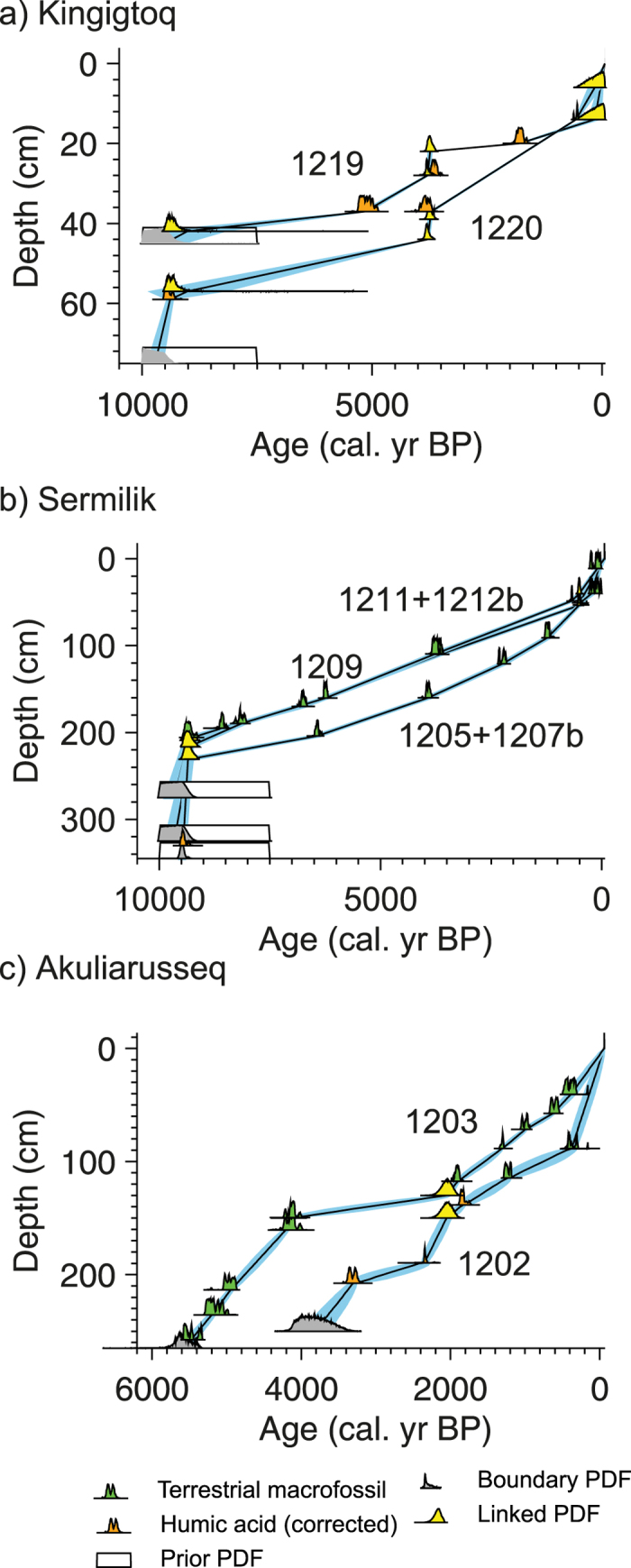
Age models for the sediment cores from Kingigtoq, Sermilik and Akuliarusseq lakes. No age models were made for Rundesø, Storesø and Kangerdluatsiaup tasia because of an insufficient number of radiocarbon ages.

Storesø and Rundesø (lakes) are located 30 km north of Narsarsuaq and were overridden when the Nordgletscher and Kiagtût sermiat (glaciers) merged and formed the Narsarsuaq moraines in the east-west orientated valley draining into Nordbosø^15^. The lakes received meltwater during the Narsarsuaq stage, but not during the LIA where the meltwater ponded in Hullet (proglacial lake) and was drained subglacially below Kiagtût sermiat into the Narsarsuaq valley^15^. Storesø is a 1.7 km long and 0.6 km wide lake and consists of several sub-basins. The core was retrieved at 14.5 m water depth (Fig. 2e). Rundesø is a circular lake with a diameter of 500 m and was cored at 9.9 m water depth (Fig. 2e). One core from each lake was analysed and they showed a similar lithology composed of two units (Fig. 5). The lower unit, unit 1, consists of laminated clay and the upper unit, unit 2, consists of gyttja. Four bulk and three macrofossil radiocarbon samples of the two cores have been dated. However, due to the inconsistent results with inverted ages (outliers) no age models have been made from the two lakes. Instead the few reliable ages are used to estimate the approximate timing of the change from clay to gyttja in the two lakes.

**Figure 7:**
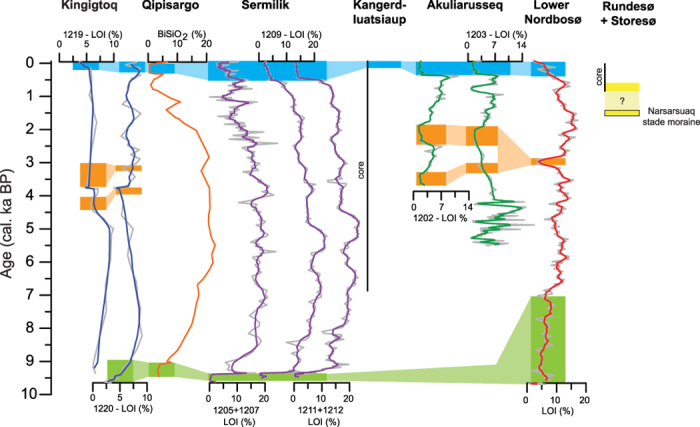
Compilation of threshold lake data from South Greenland. The horizontal bars represent periods with retracted ice margin – green: deglaciation, orange: Neoglacial, blue: LIA, yellow: Narsarsuaq stage moraine^33^. Prolonged periods of ice retreat are recorded in the central part of the QL whereas less prolonged periods of ice retreat is observed at the margin and adjacent to the QL.

The lower clay unit 1 is interpreted as representing a period of glacial meltwater input to the lake whereas the gyttja reflects a period where the ice margin was retracted behind the Narsarsuaq stage extent. In Storesø the uppermost bulk age in the clay is ~0.6 cal. ka BP and in Rundesø the transition between clay and gyttja is fixed between ~0.7 and 0.4 cal. ka BP (Fig. 5). Accordingly, meltwater flowed into the lakes from >0.9–0.6 cal. ka BP after which the ice margin retreated out of the catchment of the lakes (Fig. 7).

## Discussion

**Figure 8:**
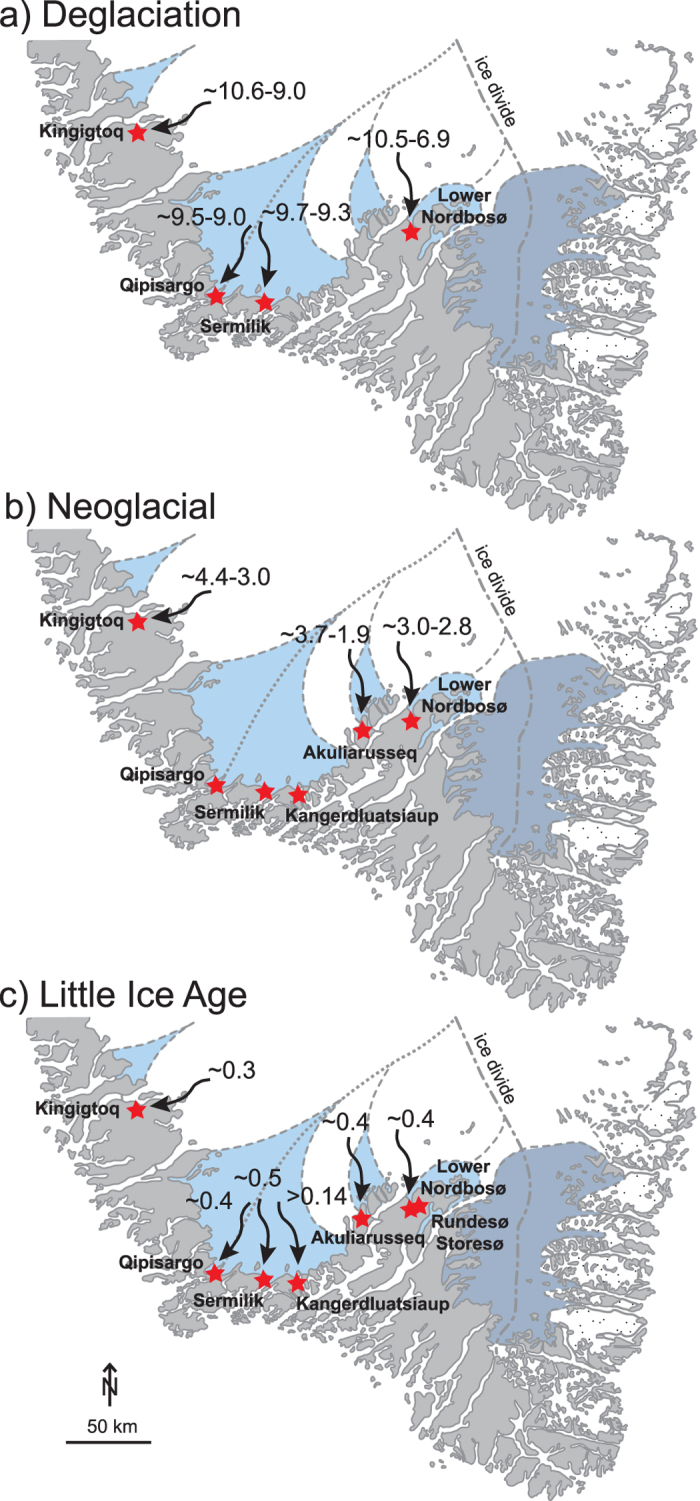
Map showing when the threshold lakes adjacent to the QL received meltwater influx during the Holocene: (**a**) Deglaciation, (**b**) Neoglacial and (**c**) LIA (Adobe Illustrator CS6 was used to create these maps; https://www.adobe.com).

The initial deglaciation is recorded in two of the six new threshold lakes showing that the area was deglaciated by ~9.6 cal. ka BP. Furthermore, the records reveal that the QL ice margin retreated outside the lake catchment areas between ~9.3–9.0 cal. ka BP (Fig. 7). This agrees well with the Qipisarqo lake record, which shows gyttja deposition starting at ~9.1 cal. ka BP^13^. It is also consistent with the ages of reworked organic material in LIA moraines, indicating that the ice margin of QL was retracted by ~9 cal. ka BP^2^. One exception is the Lower Nordbosø where the glacier remained within the catchment until ~6.9 cal. ka BP, most likely as a result of local topography rather than being governed by climate^11^. The timing of the initial ice retreat based on threshold lakes is ~1.3–1.9 ka; younger than inferred from ^10^Be surface exposure ages proximal to the LIA moraines, suggesting that the ice margin retreated within the late Holocene maximum extent by ~11.2–10.6 ka in South Greenland^12^. This discrepancy may be attributed to two issues: First, the threshold lake records are minimum limiting ages from the base of the sediment cores, which is often clay-rich and lacking dateable organic material. Second, the ^10^Be ages record when the ice retreated from the location proximal to the LIA moraines and thus it should be regarded as a maximum age of when the ice margin could have retreated behind the present extent, assuming that the early Holocene ice retreat occurred without any pauses. Accordingly, The QL ice margin reached the present extent by ~11.2–10.6 ka^12^ after which it, in most places, retreated behind the present extent during the initial deglaciation phase at minimum ~9.3–9.0 cal. ka BP (Fig. 8a).

Following the initial deglaciation, the ice margin retreated behind its current margin until the Neoglacial when the ice margin on the flanks of the QL lobe (Akuliarusseq, Lower Nordbosø) and 50 km north of the QL (Kingigtoq) reached its present extent between ~4.4 and 3.0 cal. ka BP. The ice remained at its present position from ~3.0 to1.9 cal. ka BP with a short period of ice retreat (Figs 7 and 8b). In contrast, the ice margin in the central part of the QL (Qipisargo, Sermilik, Kangerdluatsiaup tasia) remained retracted until the LIA, although it is likely that the central part of the lobe made a readvance similar to the marginal part of the lobe during the Neoglacial (Fig. 8b). A Neoglacial advance has also been recorded in threshold lakes in other areas of Greenland and the timing of the meltwater influx appear in most lakes at ~3–2 cal. ka BP^14^,^16^,^17^,^18^,^19^,^20^,^21^. However, some lakes in West and Southeast Greenland received meltwater as early as ~6.3 and ~5.0 cal. ka BP, respectively^14^,^16^. During this time the ice sheet has been shown to be smaller than present. Therefore, this seems to suggest that the ice marginal response in some places is strongly influenced by local topographic conditions rather than being governed by climate changes alone. This period of retracted ice margin coincides with the Holocene Thermal Maximum (HTM) ~8–5 cal. ka BP where the local air temperatures in South Greenland was as much as 2–4 °C warmer than present^22^,^23^ and when there was increased inflow of warm Atlantic water in East and West Greenland^24^,^25^,^26^,^27^. Recent numerical modelling based on a large number relative sea level observations from Greenland suggest that the GrIS reached a minimum Holocene ice extent ~4 ka ago and contributed with 16 cm to the global sea level^28^. This modelled ice volume minimum occurs rather late compared to the temperature maximum in Greenland, but it corresponds well with threshold lake records in southern Greenland and also suggests a significant inertia between local temperature forcing and ice sheet response^16^.

After the Neoglacial readvance that culminated between ~3 and 1.8 cal. ka BP the QL retreated in all sectors and gyttja was deposited in the lakes until ~0.5–0.2 cal. ka BP. Subsequently, the ice margin re-advanced to its LIA position (Figs 7 and 8c). The period with retracted ice margin corresponds to the last part of the Roman Warm Period (~2.25–1.6 cal. ka BP) the colder European Dark Ages (~1.6–1.25 cal. ka BP) and the Medieval Warm Anomaly (~1.25–0.75 ka cal. ka BP). However it is still unresolved how these late Holocene climate events affected local climate in Greenland. Some records show a reduced inflow of warm Atlantic water in fjords during the Roman Warm Period and the Medieval Warm Anomaly^29^, while other records show a strengthening of the of Atlantic water advection^24^,^25^,^26^,^27^,^30^ and increased atmospheric temperatures^22^,^31^ suggesting that the warmer climate most likely triggered the retracted ice margin of QL during this period. One exception is the Kiagtût Sermiat (glacier) near Narsarsuaq, which advanced outside the LIA position and formed the Narsarsuaq moraine. The age of the moraine was originally dated to ~1.2 cal. ka BP using minimum limiting radiocarbon ages of lake sediments^32^. However, a more recent study based on ^10^Be surface exposure ages suggests that Narsarsuaq moraine was formed and abandoned by 1.5 ka and the ice margin has been within or at its LIA extent since 1.3 ka^33^. These data suggest that the Kiagtût Sermiat advanced at 1.5 ka and remained close to the LIA moraine until ~0.6 cal. ka BP after which it retreated behind the LIA extent. Our results from Storesø and Rundesø also show that the Kiagtût Sermiat was retracted from c. 0.6 cal. ka BP. This behaviour is unlike the general pattern of the QL that remained retracted until ~0.5–0.2 cal. ka BP where the ice margin reached its LIA position and therefore this suggests that the Kiagtût Sermiat is not representative of the ice margin fluctuations in southern Greenland. Instead it may reflect that Kiagtût Sermiat was influenced by local topographic conditions or it may have experienced a surge-type advance, although there is no historical evidence in support of this hypothesis.

In summary, using six new threshold lake records combined with two existing threshold lake records, radiocarbon ages of reworked organic remains in LIA moraines, and published ^10^Be surface exposure ages we have constrained the Holocene glacial history of the QL – the southernmost part of the GrIS. The locations of the threshold lakes that have been analysed from various places around the QL, in conjunction to the published data, provide a unique opportunity to assess both the spatial and temporal resolution of the ice marginal changes. At the central part of the QL the ice margin was retracted from the initial deglaciation, ~9.3–9.0 cal. ka BP until the LIA ~0.5–0.2 cal. ka BP. At the margins of the QL and adjacent to the ice lobe this long period of retracted ice margin position was interrupted by a Neoglacial readvance to the present ice extent between ~4.4 and 1.9 cal. ka BP. In all sectors of the QL, the ice margin re-advanced during the LIA between ~0.5 and 0.2 cal. ka BP. These results clearly show that multiple threshold lakes may be needed to obtain a comprehensive history of ice marginal fluctuations of a larger ice lobe and that a single threshold lake record may not be sufficient to infer ice marginal fluctuations for a large part of the ice sheet. However, in general, the results show that the southern margin of the GrIS responded to the recorded Holocene climate forcings and also emphasises the role of local topography on ice marginal changes.

## Methods

We analysed sediment cores from six threshold lakes located adjacent to the present ice margin distal to the QL in South Greenland to obtain a better understanding of its Holocene ice marginal fluctuations. We chose these threshold lakes because they only receive meltwater and clastic sediments (silt and clay) during periods of close-to-present ice extent, whereas non-glacial organic rich sediments (gyttja) are deposited during intervals with restricted ice extent in the catchment^34^. This approach has successfully been adapted to study ice marginal fluctuations of the GrIS and local ice caps in Greenland^11^,^13^,^14^,^16^,^18^,^19^,^21^.

The lakes were identified using satellite and aerial photographs. In the field, an echo sounder with a built-in GPS was used to measure the lake depth and create a bathymetric map. The lakes were cored using a custom built piston corer capable of obtaining 4 m of sediments at a lake depth up to 50 m. One exception is the Kangerdluatsiaup tasia (lake) that was cored using the Uwitec coring equipment. After retrieval, the cores were kept in an upright position and drained before being packed and shipped back to the laboratory where they were stored at 2 °C. In the laboratory the cores were split in half, cleaned and a lithological description was made. An ITRAX core scanner was used to take high-resolution pictures and measure micro-XRF and magnetic susceptibility (MS) at every 2 mm and 0.5 mm, respectively. The XRF scans were made at Aarhus University core scanning facility with a molybdenum tube set at 30 kV and 30 mA with a dwell time of 30 seconds. Prior to analysis the sediment surfaces were carefully flattened and subsequently covered with thin (4 μm) ultralene film. A step size of 2 mm was selected to capture possible elemental variations in even small laminations. To illustrate the changes from gyttja to clastic sediments Ti, K or Si are often used as a measure of clastic input^11^,^35^. In this study we have used K normalized by incoherent and coherent to remove various instrumental scattering^36^. Loss-on-ignition (LOI) was analysed for every 1 cm by combustion of 2 cm^3^ sample at 550 °C for 4 hours^37^.

Samples for radiocarbon dating were sub-sampled with the primary objective to date clastic layers (meltwater) within the cores. We preferably sampled and identified terrestrial macrofossils in 1-cm intervals. When terrestrial macrofossils were absent, we used moss or bulk samples (humic acid) for radiocarbon dating. Using bulk sample to estimate the age of sediments can be problematic because bulk samples integrate carbon of both allochthonous and autochthonous origins. Consequently eroded soil carbon may for example in some cases constitute a major fraction of the bulk carbon. Hence bulk ^14^C ages therefore often deviate substantially from the true age of sediments. Further, as the origin of bulk carbon will depend on hydrology, catchment processes and morphology the bulk ^14^C age offset will most often deviate between different lakes^38^,^39^. The ^14^C bulk age offset can be estimated using paired bulk and terrestrial macrofossil samples in order to correct unpaired bulk ^14^C ages^16^. The samples were dated at the AMS ^14^C Centre at Aarhus University (AAR), Lund University (LU) and Belfast University (UBA). The age models are based on radiocarbon ages of 40 macrofossils and 18 bulk sediment samples that have been converted into calendar years using Oxcal 4.2 and calibrated to calendar years using IntCal13^40^,^41^ and are quoted as cal. yr BP or cal. kyr BP (Supplementary Data set 1). Depth-to-age models were constructed using the depositional model method of Oxcal 4.2 except for lakes Kangerdluatsiaup tasia, Rundersø and Storesø where there are too few radiocarbon ages to make a reliable age-depth model. In all age models the age of the top sediment is set to the year of coring.

## Additional Information

**How to cite this article**: Larsen, N. K. *et al*. Holocene ice marginal fluctuations of the Qassimiut lobe in South Greenland. *Sci. Rep.* **6**, 22362; doi: 10.1038/srep22362 (2016).

## Supplementary Material

Supplementary Information

## References

[b1] Kjeldsen K. K. . Spatial and temporal distribution of mass loss from the Greenland Ice Sheet since AD 1900. Nature 528, 396–400 (2015). 26672555 10.1038/nature16183

[b2] Weidick A., Kelly M. & Bennike O. Late Quaternary development of the southern sector of the Greenland Ice Sheet, with particular reference to the Qassimiut lobe. Boreas 33, 284–299 (2004).

[b3] Weidick A., Bøggild C. E. & Knudsen N. T. Glacier inventory and atlas of West Greenland. Rapp Grønlands Geol Undersøgelse 158, 1–194 (1992).

[b4] Funder S., Kjeldsen K. K., Kjær K. H. & Ó Cofaigh C. The Greenland ice sheet during the last 300.000 years: a review. Dev Quaternary Sci 15, 699–713 (2011).

[b5] Knutz P. C., Sicre M. A., Ebbesen H., Christiansen S. & Kuijpers A. Multiple-stage deglacial retreat of the southern Greenland Ice Sheet linked with Irminger Current warm water transport. Paleoceanography 26, doi: 10.1029/2010pa002053 (2011).

[b6] Winsor K., Carlson A. E., Welke B. & Reilly B. Early deglacial onset of southwestern Greenland ice-sheet retreat on the continental shelf. Quaternary Sci Rev (2015).

[b7] Carlson A., Stoner J. S., Donnelly J. P. & Hillaire-Marcel C. Response of the southern Greenland Ice Sheet during the last two deglaciations. Geology 36, 359–362 (2008).

[b8] Bennike O. & Björck S. Chronology of the last recession of the Greenland Ice Sheet. J Quaternary Sci 17, 211–219 (2002).

[b9] Winsor K., Carlson A. E., Caffee M. W. & Rood D. H. Rapid last-deglacial thinning and retreat of the marine-terminating southwestern Greenland ice sheet. Earth Planet Sc Lett 426, 1–12 (2015).

[b10] Sparrenbom C. . Holocene relative sea-level changes in the inner Bredefjord area, southern Greenland. Quaternary Sci Rev 69, 107–124 (2013).

[b11] Larsen N. K. . Restricted impact of Holocene climate variations on the southern Greenland Ice Sheet. Quaternary Sci Rev 30, 3171–3180 (2011).

[b12] Carlson A. E. . Earliest Holocene south Greenland ice sheet retreat within its late Holocene extent. Geophys Res Lett 41, 5514–5521 (2014).

[b13] Kaplan M. R., Wolfe A. P. & Miller G. H. Holocene environmental variability in southern Greenland inferred from lake sediments. Quaternary Res 58, 149–159 (2002).

[b14] Briner J. P., Stewart H. A. M., Young N. E., Philipps W. & Losee S. Using proglacial-threshold lakes to constrain fluctuations of the Jakobshavn Isbrae ice margin, western Greenland, during the Holocene. Quaternary Sci Rev 29 (2010).

[b15] Weidick A. Ice margin features in the Julianehåb District. Medd Grønland 165, 1–133 (1963).

[b16] Larsen N. K. . The response of the southern Greenland ice sheet to the Holocene thermal maximum. Geology 43, 291–294 (2015).

[b17] Young N. E. & Briner J. P. Holocene evolution of the western Greenland Ice Sheet: Assessing geophysical ice-sheet models with geological reconstructions of ice-margin change. Quaternary Sci Rev 114, 1–17 (2015).

[b18] Kelley S. E., Briner J. P., Young N. E., Babonis G. S. & Csatho B. Maximum late Holocene extent of the western Greenland Ice Sheet during the late 20th century. Quaternary Sci Rev 56, 89–98 (2012).

[b19] Young N. E. . Response of Jakobshavn Isbrae Greenland, to Holocene climate change. Geology 39, 131–134 (2011).

[b20] Håkansson L., Briner J., Andresen C. S., Thomas E. K. & Bennike O. Slow retreat of a land based sector of the West Greenland Ice Sheet during the Holocene Thermal Maximum: evidence from threshold lakes at Paakitsoq. Quaternary Sci Rev 98, 74–83 (2014).

[b21] Levy L. B. . Holocene fluctuations of Bregne ice cap, Scoresby Sund, east Greenland: a proxy for climate along the Greenland Ice Sheet margin. Quaternary Sci Rev 92, 357–368 (2014).

[b22] Frechette B. & de Vernal A. Relationship between Holocene climate variations over southern Greenland and eastern Baffin Island and synoptic circulation pattern. Clim Past 5, 347–359 (2009).

[b23] Wooller M. J. . Quantitative paleotemperature estimates from delta O-18 of chironomid head capsules preserved in arctic lake sediments. J Paleolimnol 31, 267–274 (2004).

[b24] Jennings A., Andrews J. & Wilson L. Holocene environmental evolution of the SE Greenland Shelf North and South of the Denmark Strait: Irminger and East Greenland current interactions. Quaternary Sci Rev 30, 980–998 (2011).

[b25] Jennings A. E. . Paleoenvironments during Younger Dryas-Early Holocene retreat of the Greenland Ice Sheet from outer Disko Trough, central west Greenland. J Quaternary Sci 29, 27–40 (2014).

[b26] Lloyd J. M., Kuijpers A., Long A., Moros M. & Park L. A. Foraminiferal reconstruction of mid- to late-Holocene ocean circulation and climate variability in Disko Bugt, West Greenland. Holocene 17, 1079–1091 (2007).

[b27] Telesinski M. M., Spielhagen R. F. & Lind E. M. A high-resolution Lateglacial and Holocene palaeoceanographic record from the Greenland Sea. Boreas 43, 273–285 (2013).

[b28] Lecavalier B. S. . A model of Greenland ice sheet deglaciation constrained by observations of relative sea level and ice extent. Quaternary Sci Rev 102, 54–84 (2014).

[b29] Seidenkrantz M. S. . Hydrography and climate of the last 4400 years in a SW Greenland fjord: implications for Labrador Sea palaeoceanography. Holocene 17, 387–401 (2007).

[b30] Miattinen A., Divine D. V., Husum K., Koc N. & Jennings A. Exceptional ocean surface conditions on the SE Greenland shelf during the Medival Climate Anomaly. Paleoceanography 30, doi: 10.1002/2015PA002849 (2015).

[b31] Kaufman D. S. . Recent Warming Reverses Long-Term Arctic Cooling. Science 325, 1236–1239 (2009). 19729653 10.1126/science.1173983

[b32] Bennike O. & Sparrenbom C. J. Dating of the Narssarssuaq stade in southern Greenland. Holocene 17, 279–282 (2007).

[b33] Winsor K., Carlson A. E. & Rood D. H. Be-10 dating of the Narsarsuaq moraine in southernmost Greenland: evidence for a late-Holocene ice advance exceeding the Little Ice Age maximum. Quaternary Sci Rev 98, 135–143 (2014).

[b34] Karlen W. Lacustrine sediment studies - a technique to obtain a continous record of holocene glacier variations. Geogr Ann A 63, 273–281 (1981).

[b35] Kylander M. E., Ampel L., Wohlfarth B. & Veres D. High-resolution XRF core scanning analysis of Les Echets (France) Sedimentary sequence: new insights from chemical proxies. J Quaternary Sci 26, 109–117 (2011).

[b36] Kylander M. E., Klaminder J., Wohlfarth B. & Löwemark L. Geochemical responses to paleoclimatic changes in southern Sweden since the late glacial: the Hässeldala Port lake sediment record. J Paleolimnol 50, 57–70 (2013).

[b37] Heiri O., Lotter A. F. & Lemcke G. Loss on ignition as a method for estimating organic and carbonate content in sediments: reproducibility and comparability of results. J Paleolimnol 25, 101–110 (2001).

[b38] Abbott M. B. & Stafford T. W. Radiocarbon geochemistry of modern and ancient Arctic lake systems, Baffin Island, Canada. Quaternary Res 45, 300–311 (1996).

[b39] Olsen J., Kjær K. H., Funder S., Larsen N. K. & Ludikova A. High-Arctic climate conditions for the last 7000 years inferred from multi-proxy analysis of the Bliss Lake record, North Greenland. J Quaternary Sci 27, 318–327 (2012).

[b40] Ramsey C. B. Deposition models for chronological records. Quaternary Sci Rev 27, 42–60 (2008).

[b41] Reimer P. J. . IntCal13 and Marine13 Radiocarbon Age Calibration Curves 0–50,000 Years cal BP. Radiocarbon 55, 1869–1887 (2013).

[b42] Weidick A. Gletschere i Sydgrønland, historie, natur og omgivelser. Grønlands Geol Undersøgelse , 1–80 (1988).

